# Difficulties following naturalistic psychedelic use and associations with adverse childhood experiences

**DOI:** 10.1016/j.drugpo.2025.105105

**Published:** 2025-12-13

**Authors:** Michelle Olofsson, Walter Osika, Simon B. Goldberg, Peter S. Hendricks, Predrag Petrovic, Tonya White, Cecilia U.D. Stenfors, Sankalp Chaturvedi, Otto Simonsson

**Affiliations:** a Department of Clinical Neuroscience, Karolinska Institutet, Stockholm, Sweden; b Section on Social and Cognitive Developmental Neuroscience, National Institute of Mental Health, National Institutes of Health, Bethesda, MD, USA; c Department of Neurobiology, Care Sciences and Society, Karolinska Institutet, Stockholm, Sweden; d *Department of Counseling Psychology, University of Wisconsin*–*Madison, Madison, WI, USA*; e Center for Healthy Minds, University of Wisconsin-Madison, Madison, WI, USA; f Department of Psychiatry and Behavioral Neurobiology, University of Alabama at Birmingham, Birmingham, AL, United States; g Department of Psychology, Stockholm University, Stockholm, Sweden; h Department of Management and Entrepreneurship, Imperial College London, London, UK

**Keywords:** Psychedelics, Psilocybin, Adverse, Childhood, Challenging experiences, Trauma

## Abstract

**Background:**

Naturalistic psychedelic use can result in a range of difficulties that impair social, occupational, and other important areas of functioning. Yet, the prevalence, phenomenology, and etiology of these outcomes remain poorly understood. Recent qualitative research has shown that individuals with long-term difficulties after psychedelic use sometimes attribute their challenges to childhood trauma. Further studies are needed to investigate these relationships.

**Methods:**

In this cross-sectional mixed-methods study of U.S. adults with lifetime psychedelic use (*n* = 3168), we examined the prevalence, duration, and nature of psychedelic-related difficulties, as well as associations with adverse childhood experiences (ACEs).

**Results:**

Of all participants (*n* = 3168), most (*n* = 2785, 87.9 %) reported no difficulties; 6.4 % (*n* = 203) reported post-acute difficulties that lasted for more than one day, and 1.3 % (*n* = 40) for more than one year. Among those who reported difficulties (*n* = 383), 47 % (*n* = 180) reported that their difficulties resolved in one day or less. The most frequently reported post-acute difficulties were general anxiety (33.9 %), negative changes in self-concept (25.9 %), and social disconnection (23.0 %). In covariate-adjusted regression models, 2 ACEs (aOR: 2.24, *p* = 0.007), 3 ACEs (aOR: 2.27, *p* = 0.006), and ≥4 ACEs (aOR: 2.84, *p* < 0.001) were associated with higher odds of psychedelic-related difficulties compared to 0 ACEs. ≥4 ACEs were also associated with higher odds of difficulties that lasted more than one day (aOR: 2.37, *p* = 0.015) and more than one week (aOR: 2.89, *p* = 0.042).

**Conclusion:**

There are a range of difficulties that can follow psychedelic use and childhood adversity may represent a risk factor for persistent adverse effects.

## Introduction

In recent years, there has been a resurgence in both scientific research and public interest in psychedelics, such as psilocybin and lysergic acid diethylamide (LSD; Danias & Appel, 2023; Hadar et al., 2023). Early clinical trials of psychedelic-assisted therapy have demonstrated rapid and sustained symptom reductions across various psychiatric disorders (Andersen et al., 2021), while studies in healthy volunteers and observational samples suggest psychedelics may also have broader benefits on well-being and life satisfaction (Gandy, 2019; Nayak et al., 2023; Schmid et al., 2015). In parallel, public enthusiasm for psychedelics has grown, reflected in widespread positive media coverage, increasing policy initiatives to decriminalize or legalize psychedelics, and rising rates of psychedelic use outside of research settings (Aday et al., 2020; Rockhill et al., 2025; Siegel et al., 2023). In the United States (U.S.), for instance, recent survey data suggest the number of young adults consuming psychedelics in the past year has increased by 44 % from 2019 to 2023 (Rockhill et al., 2025). Frameworks for legal psychedelic use have also already been established in Oregon and Colorado, while bills proposing similar changes in the regulatory status of psychedelics have been introduced in over 20 other U.S. states (Aday et al., 2020; Marks, 2023; Siegel et al., 2023).

Although psychedelics are suggested to pose overall fewer risks than substances such as alcohol or opioids (Nutt et al., 2010; Schlag et al., 2022), there are growing concerns that the rapid rise in psychedelic use and substantive policy changes have outpaced public health research (Evans et al., 2025; Simonsson et al., 2024; Smith & Appelbaum, 2022). While clinical studies on psychedelic-assisted therapy demonstrate promising evidence of safety and efficacy (Yao et al., 2024), these findings are unlikely to generalize to real-world contexts due to factors such as small sample sizes, careful participant selection, and highly controlled experimental conditions (Simonsson et al., 2024). Further, a recent systematic review suggested adverse effects may be underreported in clinical studies due to a lack of systematic assessment (Breeksema et al., 2022). Investigating naturalistic psychedelic use therefore represents a key research avenue to inform both harm reduction efforts and clinical safety guidelines.

While acute adverse effects of psychedelics have been widely studied, they may not necessarily indicate enduring functional impairment. Indeed, some studies have found positive associations between the degree of difficulty of the acute challenging psychedelic experience and long-term improvements in well-being (Barrett et al., 2016; Carbonaro et al., 2016). Similarly, other research has shown that people who use psychedelics sometimes report that the challenging aspects of the acute experience contribute to important life insights or play a role in positive psychological growth (Bouso et al., 2022; Gashi et al., 2021). As such, post-acute difficulties – defined as impairment in social, occupational, or other important areas of functioning that persist beyond the acute pharmacological effects – may represent a more relevant focus for psychedelic harm reduction research. Although research remains limited, prior observational studies on naturalistic psychedelic use have identified a range of potential post-acute difficulties, including anxiety, depersonalization, derealization, social disconnection, flashbacks, memory problems, persistent visual disturbances, and existential struggle (Bremler et al., 2023; Evans et al., 2023; Kopra et al., 2023; Kvam et al., 2023; Simonsson et al., 2023).

Estimates of prevalence and duration of psychedelic-related difficulties vary widely across the literature. Observational studies have found that 20–41 % of people who have used psychedelics report having experienced adverse effects due to naturalistic psychedelic use at some point in their lifetime (Kopra et al., 2023; Kvam et al., 2023; Simonsson et al., 2023). While these studies often report that such difficulties are short-lived, resolving within a few days or less, 1–4 % of people who have used psychedelics report symptoms lasting a year or longer (Kvam et al., 2023; Simonsson et al., 2023). Notably, many of these estimates are derived from highly self-selected samples (Kopra et al., 2023; Kvam et al., 2023), underscoring the need for additional research to clarify the generalizability, severity, and public health burden of psychedelic-related difficulties.

It is important to examine individual risk factors that may increase susceptibility to difficulties following naturalistic psychedelic use. One potentially relevant factor that remains understudied is adverse childhood experiences (ACEs), which are defined as potentially traumatic events that occur before the age of 18 (Felitti et al., 1998). ACEs have been most studied in the context of abuse (e.g. physical, sexual, emotional), neglect (e.g. physical, emotional), and household dysfunction (e.g. parental separation, household member incarceration, household member mental illness), but can also include other forms of early life stress such as exposure to war, natural disaster, or racial discrimination (Helton et al., 2022). While not all individuals exposed to ACEs develop chronic health problems, ACEs have been associated with increased risk of psychiatric disorders, symptom severity, and poorer response to psychiatric treatments (Copeland et al., 2018; Senaratne et al., 2024; Williams et al., 2016). As such, psychiatric patients with ACE histories may represent a key target patient group for alternative therapies such as psychedelic-assisted therapy. At the same time, individuals with ACEs may be inclined to use psychedelics in non-clinical settings. Indeed, emerging research suggests that trauma survivors are turning to psychedelics as a form of self-treatment, often outside clinical settings or through psychedelic-assisted therapy organizations outside the medical system (Glynos et al., 2024, 2023). In one survey study on U. S. residents who participated in ayahuasca ceremonies, approximately 20 % of participants cited a desire to heal childhood trauma as a primary motivation (Perkins & Sarris, 2021). Taken together, this highlights the importance of understanding the spectrum of potential benefits and risks of psychedelic use in populations with histories of childhood adversity, to inform clinical practice and minimize risk and harm in real-world use.

A small but growing body of research has examined associations between childhood adversity and naturalistic psychedelic use, yielding mixed evidence on the relationship with negative and positive outcomes. For example, observational studies have linked naturalistic psychedelic use to lower shame and complex trauma symptoms among adults with childhood maltreatment (Healy et al., 2024, 2021), and have also found associations between ACE scores and improvements in mental health (Nayak et al., 2023). Other studies have reported null findings (Cassidy et al., 2024; Mathai et al., 2025), such as no association between childhood trauma history and challenging experiences during ayahuasca use (Cassidy et al., 2024). In contrast, in a recent study investigating psychedelic-related difficulties, 40 % of participants attributed their challenges to a traumatic experience in childhood (Evans et al., 2023). A follow-up interview study revealed recurring themes such as resurfacing of traumatic memories from childhood, symbolic re-embodiment of trauma, and confusion surrounding emerging memories or emotions (Simon et al., 2025). Differences in study designs and sample characteristics may help explain these contradictory findings, but further research is needed to clarify the relationship between childhood adversity and psychedelic-related risks and harms.

In the present study, we used data from a large and diverse sample of U.S. adults (*n* = 3168) who reported a history of naturalistic psychedelic use. Through a mixed-methods qualitative and quantitative approach, we aimed to investigate the prevalence, duration, and phenomenology of psychedelic-related difficulties, as well as associations with ACEs. Building on recent research showing that some individuals with psychedelic-related difficulties attribute them to childhood trauma (Evans et al., 2023; Simon et al., 2025), we hypothesized that individuals with ACE histories would be more likely to report psychedelic-related difficulties and experience them for longer durations.

## Method

### Participants and study design

The present study used data from a larger survey project (*N* = 13,012), including only those participants who reported a history of psychedelic use (*n* = 3168; Simonsson et al., 2025). We employed a mixed methods design approach wherein both qualitative data and quantitative data were collected and analyzed in parallel (Edmonds & Kennedy, 2016; Ivankova et al., 2006). Participants were recruited through the online platform Prolific.co between June and September 2023 and restricted to U.S. residents between the ages of 18 and 50 (https://app.prolific.co; for additional details on recruitment, see Simonsson et al., 2025). The study was advertised with no specific reference to psychedelics to minimize self-selection bias. Participation in the study was monetarily incentivized with approximately $8.00 per hour in compensation. All participants who chose to participate in the study provided digital informed consent. Study procedures were determined to be exempt from the Institutional Review Board at the University of Wisconsin–Madison. We also sought an advisory opinion from the Swedish Ethical Review Authority regarding the analysis of the fully anonymized data (Dnr 2024-01208-01).

### Materials

#### Psychedelic use

Participants were asked to report which, if any, of the following psychedelics they had ever used: ayahuasca, N,N-dimethyltryptamine (DMT), LSD, mescaline, peyote, San Pedro, and psilocybin. While several substances overlap in their active ingredient (e.g. ayahuasca and DMT; mescaline, peyote, and San Pedro), they were conceptually delineated because their patterns of use, cultural contexts, and colloquial terminology differ substantially. This is in line with previous work (Simonsson et al., 2023). Those who reported psychedelic use were also asked to specify the frequency of their lifetime use.

#### Difficulties due to naturalistic psychedelic use

All participants who reported any psychedelic use in their lifetime were asked if they had ever experienced significant distress or impairment in social, occupational, or other important areas of functioning as a result of using psychedelics.

Participants who responded "yes" to this item were classified as having experienced difficulties following psychedelic use. These participants were further asked to indicate the duration of their longest experience with psychedelic-related difficulties. Responses were rated on a 5-point Likert scale: (1) "1 day or less," (2) "a few days to a week," (3) "more than 1 week to 1 month," (4) "more than 1 month to 1 year," (5) "more than 1 year". Response options were derived from a previous study investigating psychedelic-related difficulties (Simonsson et al., 2023).

Participants who reported difficulties lasting more than one day were further classified as having experienced post-acute difficulties. While individuals reporting difficulties lasting one day or less may still have experienced adverse effects beyond the acute pharmacological window, a more conservative threshold was used to define post-acute effects.

All participants were also given an opportunity to elaborate on the nature of difficulties experienced in their own words. These short responses were utilized for the qualitative analysis in this study.

#### Operationalization of adverse childhood experiences

ACEs were assessed using the short form 11-item Behavioral Risk Factor Surveillance Survey version of the ACE-Questionnaire (Centers for Disease Control and Prevention (CDC), 2010). This instrument measures exposure to adversity before the age of 18, including 5 items related to household dysfunction (i.e., parental separation/divorce, incarcerated family members, household substance abuse, domestic violence, and mental illness) and 6 items related to abuse (i.e., sexual, physical, and emotional). Exposure status for each item was rated as 0 = “No” or “Never” and 1 = “Yes” or “One or more times.” To generate a total score, items that assessed the same category of adversity were collapsed (e.g., three questions pertaining to sexual abuse and 2 questions pertaining to substance abuse), resulting in a total sum score ranging from 0 to 8 (Ford et al., 2014; Holden et al., 2020).

ACEs were further operationalized categorically as 0,1,2,3, and ≥4 ACEs. These cut-offs were determined based on previous literature that has demonstrated four or more ACEs as the highest risk group for chronic health problems (Campbell et al., 2016; Felitti et al., 1998; Wang et al., 2023). For exploratory purposes, ACEs were also analyzed using the 3 domains of household dysfunction, emotional/physical abuse, and sexual abuse, reflecting the 3-factor solution supported by prior factor analysis research (Ford et al., 2014).

#### Demographics

Participants reported their demographic characteristics, including age (recoded: 18–24, 25–34, 35–44, 45–50) and gender identity (recoded: male, female, other; see Supplemental Materials for full wording of survey items).

### Data analyses

#### Qualitative analysis

The study employed the Structured Tabular Thematic Analysis (ST-TA) method for brief text data for the qualitative analysis (Robinson, 2022). This approach leverages the calculation of theme frequency means through data organization in spreadsheet software such as Microsoft Excel. While this can be conducted in inductive and deductive forms, a hybrid approach was selected for the present study. As such, we began with a deductive (a priori) approach, informed by themes identified in existing literature on difficulties following naturalistic psychedelic use (Evans et al., 2023; Robinson et al., 2024). However, given the limited scope of prior research, this was followed by inductive (emerging) coding to allow for more flexibility and fine-tuning to this dataset. In summary, the deductive-inductive methodology followed these key steps: 1) A priori theme development; 2) Deep immersion in the data; 3) Developing revised codes and themes in context of and influenced by a priori themes; 4) Tabulating themes; 5) Checking agreement; 6) Exploring theme frequencies; 7) Thematic maps; 8) Producing the report.

Prior to theme coding, responses were screened for quality and excluded if they were too vague (lacking descriptive detail of the quality of difficulties reported), described the use of a non-psychedelic substance only, or did not meet the definition of distress or loss of functioning. 44 (11.4 %) responses were excluded from the qualitative analyses. The developed codebook and diagram of exclusions for the qualitative analyses can be found in the Supplemental Materials (Figs. S1, and S2). Further, Fisher’s exact test was used to assess group differences in theme frequencies between acute (one day or less) and post-acute (more than one day) difficulties.

Thematic coding was conducted by two analysts. Analyst 1 (WO) is a psychiatrist and researcher with experience in qualitative analysis. Analyst 2 (MO) is a PhD student with experience in psychedelic research. Qualitative results are presented in the form of a summary table of identified themes and sub-themes and a narrative report.

#### Quantitative analysis

All quantitative data analysis was performed using R version 4.3.2 (R Core Team, 2023). To evaluate whether ACEs were associated with difficulties following naturalistic psychedelic use, five covariate-adjusted logistic regression models were fitted. The primary independent variable of interest was ACEs (0,1,2,3,≥4 ACEs), and the dependent variable was difficulties due to naturalistic psychedelic use. First, a model assessing associations between ACEs and reporting any difficulties related to psychedelic use was estimated. Then, additional models were estimated to assess associations between ACEs and longer durations of post-acute difficulties, using binary outcomes defined as “more than 1 day”, “more than 1 week”, “more than 1 month”, and “more than 1 year”. All models controlled for age, gender, and frequency of lifetime psychedelic use. The sociodemographic covariates were chosen in consideration of previous literature, which has suggested differences in subjective psychedelic effects and mental health outcomes by age and gender (Kettner et al., 2019; Shadani et al., 2024). Frequency of lifetime psychedelic use was also included under the rationale that greater cumulative use of psychedelics may increase the likelihood of encountering adverse effects. A sensitivity analysis was conducted where all five models were re-estimated operationalizing the ACE-Questionnaire as a total score. As an exploratory analysis, associations between the three domains of the ACE-Questionnaire (household dysfunction, physical/emotional abuse, and sexual abuse) and psychedelic-related difficulties were also assessed.

We did not adjust for multiple comparisons. This decision was based on the relatively small number of pre-defined main models and the high conceptual overlap between the outcomes, which is often considered to make standard correction methods such as Bonferroni overly conservative (Blakesley et al., 2009). This approach is in line with recent methodological guidance that suggests adjustment for multiple comparisons may not be appropriate in such cases (Hooper, 2025).

## Results

### Descriptive statistics

A summary of the study sample characteristics stratified by prevalence of difficulties following naturalistic psychedelic use can be found in Table 1. As seen in the table, there were differences between those who reported difficulties (*n* = 383; 12.1 %) and those who did not with regard to age, gender, ACEs, and lifetime use of several substances (i.e., DMT, mescaline, LSD, ayahuasca). Additional details on the frequencies of specific ACE items can be found in the Supplemental Materials (Table S1). Of all participants, 180 (5.7 %) reported that they had difficulties that lasted one day or less, 87 (2.7 %) for a few days to one week, 45 (1.4 %) for more than one week to one month, 31 (1.0 %) for more than one month to one year, and 40 (1.3 %) for more than one year (Table 2). A comparison of the duration of difficulties across psychedelic substance groups (LSD only, psilocybin only, other psychedelics only [ayahuasca, mescaline, DMT, peyote, San Pedro], and multiple psychedelics) revealed significant differences between groups (*p* < 0.001; Tables S2-S3).

### Qualitative results

**Fig. 1.**
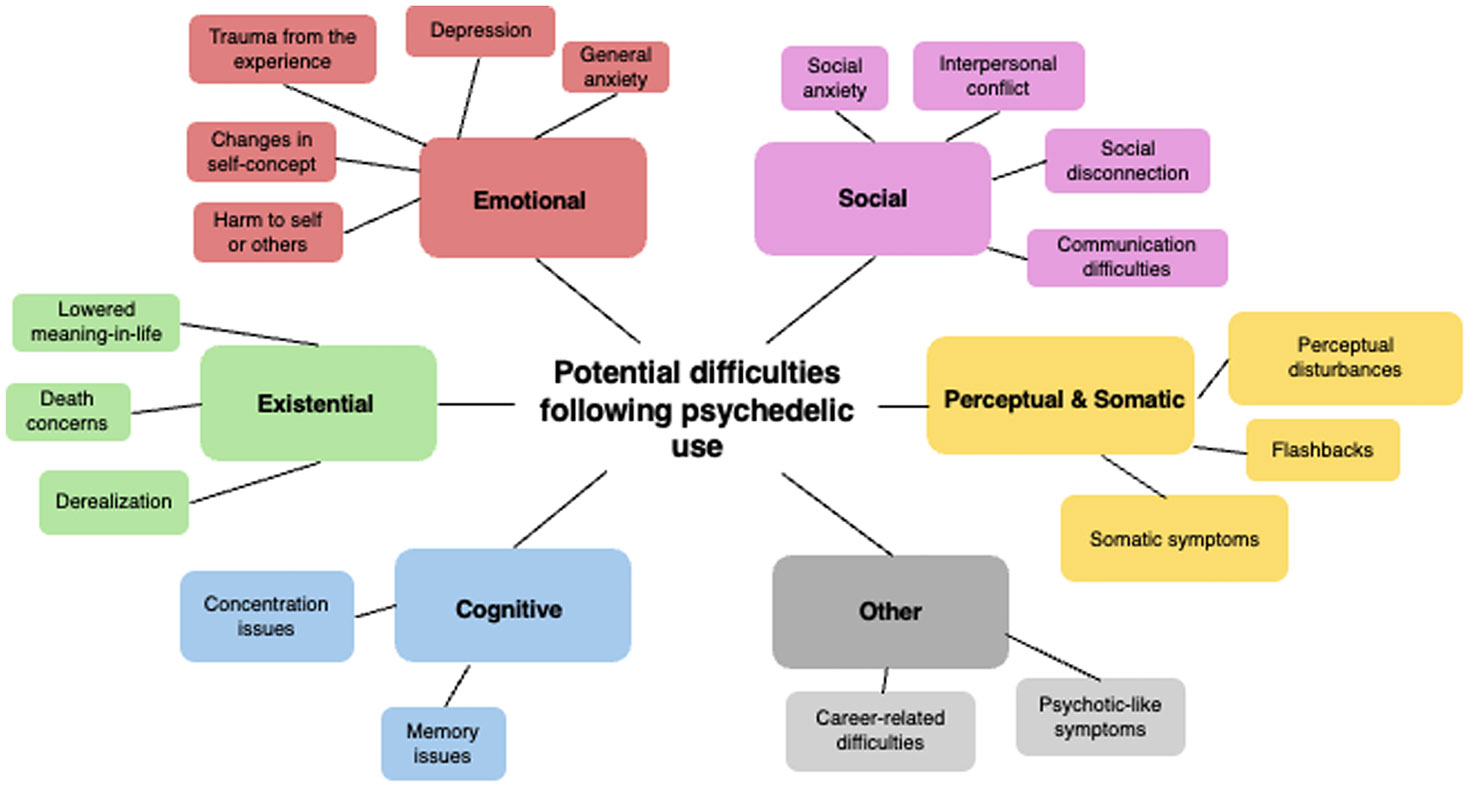
Schematic of Themes in Psychedelic-Related Difficulties. Visual summary of the final codebook of identified themes and sub-themes from the qualitative analysis.

339 participants were included for content analysis of reported difficulties. Table 3 illustrates the prevalence rates of the main themes and sub-themes. Additional details, such as theme definitions and selective illustrative quotes, can be found in the Supplemental Materials (Table S4). As shown in the table, a total of 8 main themes emerged, including emotional (83.2 %), social (53.4 %), perceptual and somatic (31.0 %), existential (20.4 %), cognitive (9.7 %), and other difficulties (13.9 %). Among sub-themes, general anxiety emerged as the most reported difficulty (43.4 %), followed by social disconnection (20.4 %), social anxiety (18.6 %), negative changes in self-concept (17.4 %), and perceptual disturbances (17.4 %). In addition, a group comparison of themes comparing acute difficulties (one day or less) and post-acute difficulties (more than one day) can be found in the Supplemental Materials (Table S5). For those who reported acute difficulties, the most common themes were general anxiety (53.3 %), social anxiety (21.2 %), perceptual disturbances (18.8 %), social disconnection (17.6 %), and somatic symptoms (14.5 %). For those who reported post-acute difficulties, the most common themes were general anxiety (33.9 %), negative changes in self-concept (25.9 %), social disconnection (23.0 %), depression (17.2 %), and career-related difficulties (16.7 %). Further, a summary of themes and sub-themes stratified by the exact duration of reported difficulties can be found in Table S6. A visual overview of the final framework of themes and sub-themes of difficulties due to psychedelic use can be found in Fig. 1. A summary of overlap in theme occurrences and differences in theme counts by substance use group (LSD only, psilocybin only, other psychedelics only [ayahuasca, mescaline, DMT, peyote, San Pedro], and multiple psychedelics) can also be found in the Supplemental Materials (Figs. S3-S4).

Given the specific research interest in post-acute difficulties, quotations from participants who reported difficulties that lasted more than one day were selected for further narrative analysis. Duration of reported difficulties for each quotation is reported in brackets.

### Emotional difficulties

Emotional difficulties emerged as the most common theme, affecting 282 (83.2 %) of participants who reported difficulties after naturalistic psychedelic use. Within this theme, several sub-themes emerged. Most commonly, feelings of heightened general anxiety were mentioned in 43.4 % of responses. These descriptions varied in intensity, from transient anxiety that resolved within a few days to more severe anxiety that resulted in the diagnosis of a psychiatric disorder and/or led to seeking professional mental health support. For instance, one participant said:

“I had intense anxiety after my trip that lasted for many months and required therapy and professional help”

(Reported duration: for more than one year)

In addition, descriptions of increased depressive symptoms were mentioned by 43 (12.7 %) participants. Responses covered themes such as low mood, low motivation, hopelessness, or sadness in words such as:

“The feeling I could remember while being under the influence left a lasting impression. It was a dark, empty, soul-crushing feeling.”

(Reported duration: for a few days to one week)

For 59 (17.4 %) participants, difficulties related to negative changes in self-concept were reported. Many described experiences of obsessive rumination over identity and self-worth, with themes suggestive of guilt or shame over past actions. For example:

“I obsessed over every failing I’ve ever had”

(Reported duration: for a few days to one week)

Other responses related to negative changes in self-concept were characterized by themes of depersonalization, defined as feeling detached one’s sense of self. For instance:

“I was generally jumpy, hypervigilant, scared, but detached or depersonalized from myself for a couple weeks. I didn’t feel real, so when things happened around me, I was terrified.”

(Reported duration: for a few days to one week)

30 (8.8 %) participants reported experiences suggestive of being traumatized from the psychedelic experience. One participant shared:

“The only time I took LSD I had an extremely bad trip. I thought I died, and the people I was with convinced me my trip was real. They did not try to help me and instead made things significantly worse. I lost a friend because of the experience and would have visceral distress and near PTSD level sensations whenever someone would bring up taking psychedelics for over a year.“

(Reported duration: for more than one year)

Of note, 3 (0.9 %) participants who had difficulties reported thoughts of harm to self or others due to their psychedelic experience. For example:

“I was unable to process conversations with friends, perceived strangers as expressing negative opinions about me, and eventually attempted suicide due to a perceived lack of value to the world at large and my friends in specific.”

(Reported duration: for a few days to one week)

### Social difficulties

Social difficulties were the second most reported main theme, occurring in 181 (53.4 %) participant responses. Within this theme, social disconnection was the most reported sub-theme (*n* = 69; 20.4 %). Some participants attributed their social disconnection to feeling as though others did not understand them after a psychedelic experience. For instance, one participant remarked:

“I felt that I was just vibrating and connecting with everything on a different level and others just did not understand because we were not on the same wave length. This caused me to become a recluse because it felt weird to be around other people.”

(Reported duration: for more than one month to one year)

Anxiety specific to socializing was mentioned by 63 (18.6 %) participants. While this theme often overlapped with social disconnection, social anxiety was distinct in its characteristics of worry in social situations. As with the theme of general anxiety, the intensity of social anxiety varied from transient experiences that were explicitly mentioned as resolving within a few days to more long-lasting difficulties, as illustrated here:

“That same trip really completely shattered me. People looked different, I felt different. I’ve had problems with social anxiety ever since then really.”

(Reported duration: for more than one year)

For 25 (7.4 %) participants, interpersonal conflict as a result of psychedelic use was mentioned. Participants cited changes in their perspective of relationships, such as:

“I felt very suspicious of my friends and their motives, our relationship has not been the same since.”

(Reported duration: a few days to one week)

In addition, difficulties specific to communication were described by 24 (7.1 %) participants. Challenges with articulation and feeling understood by others were described in words such as:

“*I was unable to speak correctly.”*

(Reported duration: for a few days to one week)

### Existential difficulties

For 69 (20.4 %) participants, difficulties covered themes existential in nature. For 25 (7.4 %) participants, this included reflections of distress related to meaning in life, sense of purpose, and abstract or philosophical anxieties related to willpower. For instance:

“It made me realize that if it’s up to me how I want to frame reality then I’m not sure I even have the willpower.“

(Reported duration: for a few days to one week)

Secondly, 25 (7.4 %) participants described feelings of unease specific to the nature of reality, typically expressing uncertainty about whether they were awake, alive, or if the world around them was “fake”. These were coded as derealization. It should be noted that this theme often co-occurred with depersonalization, where participants felt disembodied from their sense of self. Below is an example of derealization:

“I felt extremely out of it and super derealized and depersonalized for about 3 weeks. During that time I felt like nothing was real even though I knew logically it was.”

(Reported duration: for more than one week to one month)

Lastly, 19 (5.6 %) participants mentioned existential difficulties specific to death. This included some responses in which participants were uncertain if they were alive, and other responses where individuals developed a long-term fear of death. For instance:

“It gave me an existential crisis which led to having a fear of death for AT LEAST 3 years that I can confidently say where my every waking hour I was consumed with the thought of dying.”

(Reported duration: for more than one year)

### Perceptual and somatic difficulties

Difficulties related to changes in perceptual and somatic functioning were mentioned by 105 (31.0 %) participants, of which perceptual disturbances were most common (*n* = 59; 17.4 %). It should be noted that for many, perceptual changes resolved within a few days of the experience and were not ascribed as a primary cause of distress. For example, one participant mentioned:

“I was seeing colors weird for a few days after.”

(Reported duration: for a few days to one week)

However, other participants had prolonged perceptual disturbances, potentially suggestive of Hallucinogen Persisting Perceptual Disorder (Martinotti et al., 2018). An example can be seen here:

“I had to go to rehab and I still see visuals on the daily”

(Reported duration: for more than one year)

In addition, 13 (3.8 %) participants reported flashbacks – which were defined as descriptions of sudden or involuntary occurrences of a memory. Most flashbacks were related to memories from a psychedelic experience. These were generally described as frightening, and in some cases occurred in unsafe environments, such as in this quotation:

*“I was nervous to drive for many years after since I had a flashback while driving. It’s been nine years since the experience and only in the last couple of years do I feel that I have recovered from the experience*.”

(Reported duration: for more than one year)

Notably, one participant described flashbacks of forgotten childhood memories:

“I had strange flashbacks randomly starting days after the trip, they were memories from my childhood that I had forgotten.”

(Reported duration: for more than one week to one month)

Somatic difficulties also came up in several reports (9.7 %), including symptoms such as vomiting, nausea, migraines, constipation, overheating, sleep disturbances, changes in appetite, a feeling of loss of control over motor function, and sensitivity to light. For instance:

“After, I became incredibly sensitive to light. That lasted for about three days.”

(Reported duration: for a few days to one week)

### Cognitive difficulties

33 (9.7 %) participants reported cognitive difficulties after psychedelic use. Several participants reported concentration issues (*n* = 25; 7.4 %), which were often described as “brain fog” or feeling cognitively slower. For instance:

“*For a few days after use I was having a hard time keeping up with conversations. It felt as if my brain had slowed down to half speed and everyone else’s was going too fast to understand.”*

(Reported duration: for a few days to one week)

8 (2.4 %) participants reported memory issues, such as uncertainty about the accuracy of memories, or concerns with memory loss, as seen here:

“I later was diagnosed with severe PTSD and had even seen a neurologist because I was having large gaps in my memory. Forgetting who I am and where I have been.”

(Reported duration: for more than one month to one year)

### Other difficulties

Among other difficulties that did not fit within the defined main themes, career-related difficulties were reported by 30 (8.8 %) participants. Participants commonly reported challenges such as reduced job performance and loss of motivation. One example can be seen here:

“I had a lot of work anxiety and did not want to get on calls or go back into my office. Extreme dread.”

(Reported duration: for a few days to one week)

17 (5.0 %) participants mentioned symptoms suggestive of psychotic-like symptoms such as delusional ideation, disorganized thinking, and impairment in distinguishing reality from imagination. For instance:

“Felt like I was losing my mind because I thought I was telepathic.”

(Reported duration: for more than one week to one month)

### Contrasting cases

It should be noted that 4 (1.2 %) participants explicitly mentioned positive effects co-occurring with difficulties due to a psychedelic experience. Responses included themes such as bliss and peace. For example:

“To clarify it was several, “flashbacks” so to speak…lasting maybe 15 min each…heightened panic feeling, hyper alertness, paranoia…though the trip itself had a horrible aspect…the blissful aspect was even greater”

(Reported duration: for one day or less)

### Quantitative associations between ACEs and difficulties following psychedelic use

Table 4 displays the logistic regression models illustrating associations between ACEs (1,2,3, ≥4) and psychedelic-related difficulties, relative to 0 ACEs. In covariate-adjusted regression models, 2 ACEs (aOR: 2.24, 95 % CI: [1.27,4.16], *p* = 0.007), 3 ACEs (aOR: 2.27, 95 % CI: [1.29,4.20], *p* = 0.006), and ≥4 ACEs (aOR: 2.84, 95 % CI: [1.72,5.03], *p* < 0.001) were associated with higher odds of reporting any difficulties following psychedelic use. No association was found between 1 ACE (aOR: 1.33, 95 % CI: [0.71,2.58], *p* = 0.378) and reporting of any difficulties following psychedelic use. When the outcome was defined as difficulties with a duration of more than one day (i.e. post-acute difficulties), only ≥4 ACEs (aOR: 2.37, 95 % CI: [1.25,5.12], *p* = 0.015) remained associated with higher odds of reporting psychedelic-related difficulties. When models were fit for longer durations of psychedelic-related difficulties, ≥4 ACEs were associated with post-acute difficulties for more than one week (aOR: 2.89, 95 % CI: [1.17,9.63], *p* = 0.042). No association was observed between ≥4 ACEs and post-acute difficulties for more than one month or one year. 2 ACEs and 3 ACEs showed no association with reporting difficulties that lasted more than one day, more than one week, more than one month, or more than one year. The full model outputs can be found in the Supplemental Materials (Table S7). In sensitivity analyses where ACEs were coded as a continuous total score, a higher ACE score was associated with higher odds of reporting any difficulties (aOR; 1.13, 95 %CI: [1.08,1.19], *p* < 0.001; see Supplemental Materials, Table S8). Associations were also observed between ACE total score and difficulties lasting more than one day (aOR: 1.12; 95 % CI: [1.05,1.20], *p* < 0.001), more than one month (aOR: 1.17; 95 % CI: [1.05,1.31], *p* = 0.004), and more than one year (aOR: 1.17; 95 % CI: [1.01,1.35], *p* = 0.033). No association was observed between total ACE score and difficulties for more than one week (aOR: 1.09; 95 % CI [1.00, 1.19], *p* = 0.052). A cross-tabulation of the frequencies of duration of difficulties by ACE group can also be found in the Supplemental Materials (Table S9). An exploratory analysis assessing associations between the three domains of the ACE-Questionnaire showed associations between sexual abuse and reporting any difficulties (aOR: 1.43; 95 % CI: [1.12,1.83], *p* = 0.005), difficulties that lasted more than one day (aOR: 1.95; 95 % CI: [1.41,2.68], *p* < 0.001), more than one week (aOR: 2.54; 95 % CI: [1.68,3.84], *p* < 0.001), and more than one month (aOR: 2.36; 95 % CI: [1.40,3.97], *p* = 0.001). Associations were also observed between physical/emotional abuse and any difficulties (aOR: 1.64; 95 % CI: [1.23,2.23], *p* = 0.001) and difficulties lasting more than one day (aOR: 1.69; 95 % CI: [1.14,2.58], *p* < 0.001). No associations were observed between household dysfunction and psychedelic-related difficulties (Table S10).

## Discussion

The present study investigated the prevalence, duration, and nature of difficulties following naturalistic psychedelic use, as well as associations with childhood adversity. In our sample of 3168 U.S. adults with a history of psychedelic use, 383 (12.1 %) participants reported experiencing difficulties in social, occupational, or other important areas of functioning following psychedelic use at least once in their lifetime. Among those who reported difficulties, nearly half indicated that their difficulties resolved within one day or less. 6.4 % of all study participants reported having experienced post-acute difficulties after psychedelic use. Notably, 40 (1.3 %) participants reported that their difficulties lasted for more than one year. These figures broadly correspond with results from prior studies, in which approximately 9 % of participants who had used psychedelics reported difficulties that lasted one day or longer (Simonsson et al., 2023) and 1–4 % for one year or longer (Kvam et al., 2023; Simonsson et al., 2023). Given rising rates of naturalistic psychedelic use in the U.S. (Rockhill et al., 2025), even a relatively low prevalence of 1 % of people who use psychedelics experiencing difficulties for over one year could translate into hundreds of thousands of cases in the U.S. alone. This highlights the importance of further research on the adverse effects of naturalistic psychedelic use.

Thematic analysis of participants’ experiences further revealed a wide range of intensity and nature of psychedelic-related difficulties. A total of eight main themes emerged - emotional (83.2 %), social (53.4 %), perceptual and somatic (31.0 %), existential (20.4 %), cognitive (9.7 %), and other difficulties (13.9 %). Among sub-themes, general anxiety emerged as the most reported difficulty (43.4 %), followed by social disconnection (20.4 %), social anxiety (18.6 %), negative changes in self-concept (17.4 %), and perceptual disturbances (17.4 %). This is generally in line with previous observational studies of psychedelic-related difficulties, which have notably also identified anxiety as the most common theme (Argyri et al., 2025; Bouso et al., 2012; Evans et al., 2023; Simonsson et al., 2023). It is worth noting that, in contrast to previous qualitative research on psychedelic-related difficulties, we developed a codebook with a reduced number of themes from 60 to 19 (Evans et al., 2023). While this approach may be less nuanced, the shorter codebook offers a more interpretable and replicable coding system for future research.

An outstanding question in research on post-acute psychedelic difficulties is the mechanisms through which psychedelic use may lead to enduring functional impairments. A closer look at the lived experiences of participants in the present study may provide some insight. First, several participants described persistent emotional distress related to the acute psychedelic experience, noting how they could not shake feelings of intense distress, with some participants explicitly stating traumatization. These narratives may be relevant to the growing discussion of the risks of ‘traumatic psychedelic experiences’, where frightening or horrific feelings during a psychedelic experience can lead to trauma-related psychopathology (Calder et al., 2025). Second, another potentially relevant mechanism of post-acute psychedelic difficulties may be ‘ontological shock’, a phenomenon in which individuals are confronted with an overwhelming, sudden change in previous worldviews or beliefs (Argyri et al., 2025). From this perspective, the therapeutic capacity of psychedelics to induce rapid cognitive restructuring or “accommodation” of prior beliefs can also cause cognitive dissonance and manifest as mental distress (Hendricks, 2018). In the present study, participants often described distress related to sudden changes in beliefs about meaning in life, their concept of self, and social relationships. Third, several participants described how a heightened sense of connection after psychedelic use led them to feel misunderstood and disconnected from others in their daily life. This may highlight the importance of the “matrix”, a concept that has been described as a third critical factor alongside “set” and “setting” (Eisner, 1997). The “matrix” refers to the broader social and environmental context an individual comes from and returns to after psychedelic use. In the context of the present study’s participant narratives, several individuals described post-acute distress rooted in a mismatch between their newfound perceptions of connection and a lack of understanding from their everyday social environment, suggesting that the “matrix” may be an important predictor in long-term outcomes. Taken together, the examples from the present study may highlight how qualities of the psychedelic experience could be profound or therapeutically meaningful for some, yet destructive and damaging for others. These findings urge future research to investigate how individual variation in responses to acute psychedelic effects may shape long-term functioning.

The primary quantitative finding in this study was an association between ACEs and psychedelic-related difficulties. These results broadly aligns with findings from a recent study in which 40 % of people who experienced difficulties after naturalistic psychedelic use attributed their challenges to a traumatic experience in childhood (Evans et al., 2023; Simon et al., 2025). While causality cannot be definitively established from these observational studies, several potential mechanisms may explain the association between ACEs and psychedelic-related difficulties. First, extensive research has linked ACEs with increased risk of psychiatric disorders, as well as differences in social and mental functioning, such as heightened emotional reactivity and neuroticism (Bomysoad & Francis, 2020; Felitti et al., 1998; Grusnick et al., 2020; Tzouvara et al., 2023). Accordingly, the observed relationship may reflect ACEs as a general vulnerability factor for adverse psychological outcomes. Given that psychedelics can invoke intense and challenging emotional states, individuals with a history of ACEs may be more prone to both adverse reactions and experiencing such states as distressing due to pre-existing psychological vulnerabilities. Alternatively, ACEs may represent a psychedelic-specific risk. Psychedelics have been suggested to increase autobiographical recall (Healy, 2021), and there is growing discussion of the resurfacing of forgotten or repressed traumatic memories as a result of psychedelic use (Calder et al., 2025; Simon et al., 2025). Individuals who have reported childhood trauma re-experiencing under the influence of psychedelics outside of clinical settings have described the experiences as overwhelming and frightening, sometimes exhibiting symptoms suggestive of re-traumatization (Bremler et al., 2023). In a recent interview study of people who had used psychedelics and attributed adverse effects to childhood trauma, three main themes emerged: resurfacing of traumatic memories from childhood, symbolic re-embodiment of their trauma, and confusion about memories or feelings that surfaced (Simon et al., 2025). This uncertainty about the accuracy of memories that were recalled during psychedelic use highlights a larger concern in psychedelic research, which is the risk of false memories or insights (McGovern et al., 2024). Future research is needed to clarify whether the present finding reflects ACEs as a general psychological vulnerability factor or whether psychedelic-specific risks, such as re-traumatization, are a significant concern amongst adults with childhood trauma.

These findings may also be relevant in the context of clinical research on psychedelic-assisted therapies. Several documented cases from clinical trials describe patients experiencing vivid recollections or re-experiencing of traumatic memories from childhood (Malone et al., 2018; Noorani et al., 2018; Swift et al., 2017; Watts et al., 2017). While some individuals report these experiences as therapeutically meaningful, the importance of strong psychological support in coping with the distressing material has been emphasized by patients and medical professionals (Glynos et al., 2023; Noorani et al., 2018; Timmermann et al., 2022). These findings underscore the importance of future research into both the risks and benefits of psychedelic use among individuals with a history of ACEs, particularly in unregulated settings with minimal guidance or support. Clinically, these findings may translate to a need for enhanced screening of patient history of potentially traumatic experiences, trauma-informed preparation, or specialized integration support.

### Limitations

The present research has several limitations that warrant careful consideration when interpreting the findings. First, the cross-sectional design of this study limits our ability to establish causality between ACEs and difficulties following naturalistic psychedelic use. For example, it is possible that individuals who experience distressing psychedelic experiences may be more likely to recall or reinterpret their childhood experiences as traumatic (Rose, 2024). Recall bias is also a concern, as both ACE history and post-acute difficulties were self-reported, and retrospective reporting of difficult or traumatic events may be influenced by these often being repressed or forgotten over time.

Second, this study also did not assess contextual factors of psychedelic use, such as dose, physical environment, intent of use, or psychological support. The context in which psychedelics are taken is commonly described as the “set” and “setting,” which studies have suggested influence the psychological effects of psychedelics (Borkel et al., 2024; Golden et al., 2022). Given that previous research has shown associations of factors such as high dose, a lack of psychological support, use at a younger age, and pre-existing psychiatric conditions with adverse reactions, this may represent an important overlooked confounding factor (Bouso et al., 2022; Simonsson et al., 2023). In relevance to the present investigation, it is possible that individuals who have experienced more ACEs are more likely to use psychedelics at a younger age, or in an environment with less psychological support or riskier contexts, which could in part account for elevated rates of post-acute difficulties. As such, a more detailed assessment of set, setting, and dose in future research should be considered to clarify the contribution of ACEs from contextual risks.

Third, it was not the purpose of this study to assess whether participants had experienced positive changes in social, occupational, or other important areas of functioning following psychedelic use. The presence of post-acute difficulties does not preclude the possibility that some individuals who experienced post-acute difficulties also experienced long-term psychological benefits from psychedelics, as observed in previous research (Card et al., 2023; Healy et al., 2024; Nayak et al., 2023). It is also possible that some individuals may interpret the adverse mental health effects of psychedelic use as part of a positive therapeutic process, as observed in previous research (Bouso et al., 2022; Gashi et al., 2021). Such participants in the current study may not have endorsed experiencing psychedelic-related difficulties, yet may have experienced overlapping themes of adverse effects. To develop a more nuanced understanding of relationships between ACEs and psychedelic use, future research should consider assessing both adverse and positive mental health outcomes concurrently.

Fourth, associations between ACEs and psychedelic-related difficulties may be moderated by protective factors. Although past literature has demonstrated associations between ACEs and negative mental health outcomes, not all individuals who experience ACEs develop long-term health problems. For instance, the presence of strong social support, safe neighborhoods, and other positive childhood experiences have been shown to predict resilience to poor mental health outcomes (Cheong et al., 2017; Moore & N. Ramirez, 2016; Samji et al., 2024). As the current study did not assess for protective factors, this is an important topic for future research.

Finally, in its reliance on one open-ended, fill-in-the-blank item, the qualitative component of this study was limited in capturing the full phenomenology of psychedelic-related difficulties. Specifically, the current study lacked the comprehensive and thorough querying and assessment needed to explore the etiology of difficulties and their potential relationship with ACEs, such as the resurfacing of traumatic memories. Future research would benefit from employing more in-depth questionnaires or structured interviews that specifically target these themes in greater detail.

## Conclusions

As rates of naturalistic psychedelic use continue to rise, characterizing potential risks is increasingly relevant. While clinical research has highlighted therapeutic potential of psychedelics in controlled clinical settings, less is known about adverse effects in real-world contexts. Notably, this study found an association between exposure to adversity in childhood and a higher risk of difficulties following psychedelic use. Although this study cannot establish causality, these results underscore the importance of considering trauma history in the study of psychedelic use, both to enhance harm reduction strategies and to inform clinical research regarding psychedelics for populations with histories of ACEs.

## Supplementary Material

Supplementary material

Supplementary material associated with this article can be found, in the online version, at doi:10.1016/j.drugpo.2025.105105.

## Figures and Tables

**Table 1:** Sample Characteristics. Selected sample characteristics stratified by endorsement of psychedelic difficulties.

Characteristic	Total Sample *N* = 3168^a^	No Psychedelic Difficulties *N* = 2,785^1^	Psychedelic Difficulties *N* = 383^1^	*p*- value^b^
**Age**				**<0.001**
18–24	588 (18.6 %)	493 (17.7 %)	95 (24.8 %)	
25–34	1410 (44.5 %)	1223 (43.9 %)	187 (48.8 %)	
35–44	846 (26.7 %)	766 (27.5 %)	80 (20.9 %)	
45–50	324 (10.2 %)	303 (10.9 %)	21 (5.5 %)	
**Gender**				**0.006**
Male	1312 (41.4 %)	1125 (40.4 %)	187 (48.8 %)	
Female	1638 (51.7 %)	1468 (52.7 %)	170 (44.4 %)	
Other	218 (6.9 %)	192 (6.9 %)	26 (6.8 %)	
**ACE Score**				**<0.001**
0	286 (9.0 %)	270 (9.7 %)	16 (4.2 %)	
1	386 (12.2 %)	357 (12.8 %)	29 (7.6 %)	
2	443 (14.0 %)	390 (14.0 %)	53 (13.8 %)	
3	463 (14.6 %)	409 (14.7 %)	54 (14.1 %)	
4	501 (15.8 %)	432 (15.5 %)	69 (18.0 %)	
5	395 (12.5 %)	334 (12.0 %)	61 (15.9 %)	
6	320 (10.1 %)	285 (10.2 %)	35 (9.1 %)	
7	252 (8.0 %)	211 (7.6 %)	41 (10.7 %)	
8	122 (3.9 %)	97 (3.5 %)	25 (6.5 %)	
**DMT**	470 (14.8 %)	379 (13.6 %)	91 (23.8 %)	**<0.001**
**Mescaline**	219 (6.9 %)	172 (6.2 %)	47 (12.3 %)	**<0.001**
**Peyote**	123 (3.9 %)	105 (3.8 %)	18 (4.7 %)	0.458
**Psilocybin**	2468 (77.9 %)	2167 (77.8 %)	301 (78.6 %)	0.780
**LSD**	2077 (65.6 %)	1785 (64.1 %)	292 (76.2 %)	**<0.001**
**Ayahuasca**	93 (2.9 %)	72 (2.6 %)	21 (5.5 %)	**0.003**
**San Pedro**	60 (1.9 %)	49 (1.8 %)	11 (2.9 %)	0.194
**Frequency of Psychedelic Use**				**<0.001**
1	441 (13.9 %)	417 (15.0 %)	24 (6.3 %)	
2–5	1182 (37.3 %)	1062 (38.1 %)	120 (31.3 %)	
6–10	608 (19.2 %)	531 (19.1 %)	77 (20.1 %)	
11–20	431 (13.6 %)	364 (13.1 %)	67 (17.5 %)	
21–50	291 (9.2 %)	237 (8.5 %)	54 (14.1 %)	
51–100	118 (3.7 %)	99 (3.6 %)	19 (5.0 %)	
101–300	61 (1.9 %)	48 (1.7 %)	13 (3.4 %)	
300+	36 (1.1 %)	27 (1.0 %)	9 (2.3 %)	

a n (%).

b Pearson’s Chi-squared test.

**Table 2:** Duration of Psychedelic-Related Difficulties. Describes the frequency of reported duration of difficulties and the relative proportion (%) of the total sample of participants and the sub-sample of participants who reported psychedelic-related difficulties.

Duration of Psychedelic- Related Difficulties	n	% of Total Sample (*n* = 3168)	% of Difficulties Sample (*n* = 383)
Any duration	383	12.1 %	—
≤ 1 day	180	5.7 %	47.0 %
∣> *a* few days	203	6.4 %	53.0 %
> 1 week	116	3.7 %	30.3 %
> 1 month	71	2.2 %	18.5 %
> 1 year	40	1.3 %	10.4 %

**Table 3:** Frequencies of Themes and Sub-Themes. Descriptive summary of themes and sub-themes from qualitative analysis of included free text responses describing psychedelic-related difficulties.

Theme	Sub-theme	*N* = 339
**Emotional difficulties**		282 (83.2 %)
	*General anxiety*	147 (43.4 %)
	*Depression*	43 (12.7 %)
	*Negative* c*hanges in self-concept*	59 (17.4 %)
	*Trauma from the experience*	30 (8.8 %)
	*Harm to self or others*	3 (0.9 %)
**Social difficulties**		181 (53.4 %)
	*Social anxiety*	63 (18.6 %)
	*Social disconnection*	69 (20.4 %)
	*Interpersonal conflict*	25 (7.4 %)
	*Communication difficulties*	24 (7.1 %)
**Existential difficulties**		69 (20.4 %)
	*Lowered meaning in life*	25 (7.4 %)
	*Death concerns*	19 (5.6 %)
	*Derealization*	25 (7.4 %)
**Perceptual & Somatic difficulties**		105 (31.0 %)
	*Perceptual disturbance*	59 (17.4 %)
	*Flashbacks*	13 (3.8 %)
	*Somatic symptoms*	33 (9.7 %)
**Cognitive difficulties**		33 (9.7 %)
	*Memory issues*	8 (2.4 %)
	*Concentration issues*	25 (7.4 %)
**Other difficulties**		47 (13.9 %)
	*Psychotic-like symptoms*	17 (5.0 %)
	*Career-related difficulties*	30 (8.8 %)

**Table 4:** Associations Between ACEs and Psychedelic-Related Difficulties. Each column represents a unique model defined by the duration of psychedelic-related difficulty reported (i.e. any difficulties, > 1 day, > 1 week, > 1 month, >1 year). Covariates include age, gender, and frequency of lifetime psychedelic use. Adjusted odds ratios (aOR) with 95 % confidence intervals (CI) are reported for each ACE category, with statistically significant *p*-values (<0.05) in bold.

	Any Difficulties			> 1 day			> 1 week			> 1 month			> 1 year		
Characteristic	aOR	95 % CI	p- value	aOR	95 % CI	p- value	aOR	95 % CI	p- value	aOR	95 % CI	p- value	aOR	95 % CI	*p*- value
ACEs															
0	—	—		—	—		—	—		—	—		—	—	
1	1.33	0.71, 2.58	0.378	1.44	0.65, 3.42	0.385	2.72	0.97, 9.66	0.080	2.93	0.72, 19.6	0.177	2.22	0.28, 45.2	0.491
2	2.24	1.27, 4.16	**0.007**	1.97	0.94, 4.52	0.086	2.43	0.87, 8.59	0.118	2.41	0.59, 16.1	0.270	2.96	0.47, 57.2	0.324
3	2.27	1.29, 4.20	**0.006**	2.14	1.03, 4.88	0.053	2.15	0.75, 7.68	0.185	2.46	0.61, 16.5	0.259	3.67	0.62, 69.9	0.232
≥4	2.84	1.72, 5.03	**<0.001**	2.37	1.25, 5.12	**0.015**	2.89	1.17, 9.63	**0.042**	3.89	1.18, 24.0	0.063	4.36	0.90, 78.5	0.152

## Data Availability

Data and code can be shared upon reasonable request.
