# Supplementary material for: Difficulties following naturalistic psychedelic use and associations with adverse childhood experiences
Source: Int J Drug Policy. Author manuscript; Available in PMC 2026 Jan 3. (PMC12758006; doi:10.1016/j.drugpo.2025.105105)
Supplement: Supplementary material [file NIHMS2130555-supplement-Supplementary_material.docx]

### Supplemental Materials

#### Gender identity questionnaire item

What is your gender?

- Male
- Female
- Transgender (male to female)
- Transgender (female to male)
- Non-binary gender
- Other

#### Age questionnaire item

What is your age?

#### Lifetime substance use questionnaire

In your life, which of the following drugs have you EVER used? (select all that apply)

- Ayahuasca
- DMT
- LSD, also called ‘acid’
- Mescaline
- Peyote
- Psilocybin, also called ‘magic mushrooms’
- San Pedro
- Alcohol
- Nicotine products (e.g., cigarettes, e-cigarettes, cigarillos, little cigars, smokeless tobacco)
- Cannabis products (e.g., weed, THC, CBD, hemp oil)
- MDMA, also called 'ecstacy'
- Major stimulants (e.g., cocaine, methamphetamine)
- Illicit narcotic analgesics/opioids (e.g., morphine, heroin, oxycodone)
- Illicit benzodiazepines and barbiturates (e.g., Valium, Alprazolam [Xanax])
- Inhalants (poppers, whip-its, nitrous oxide, glue)
- Other substances
- None of the above

### Psychedelics frequency questionnaire

In your lifetime, how many times have you used psychedelics?

- 1
- 2-5
- 6-10
- 11-20
- 21-50
- 51-100
- 101-300
- More than 300 times

#### Difficulties after psychedelic use questionnaire

Have you ever experienced significant distress or impairment in social, occupational, or other important areas of functioning as a result of using psychedelics?

- Yes
- No

#### Duration of difficulties after psychedelics use questionnaire

How long did the experience of significant distress or impairment in social, occupational, or other important areas of functioning as a result of using psychedelics last? If you had several such experiences, please pick the response that corresponds with the experience that lasted the longest.

- 1 day or less
- For a few days to 1 week
- More than 1 week to 1 month
- More than 1 month to 1 year
- More than 1 year
- I have NEVER had such an experience as a result of using psychedelics

#### Open text response regarding difficulties after psychedelics questionnaire

Please describe the experience of significant distress or impairment in social, occupational, or other important areas of functioning as a result of using psychedelics.

**Figure S1. Qualitative theme codebook.** Excel spreadsheet outlining the final defined themes and sub-themes used in qualitative anlaysis.


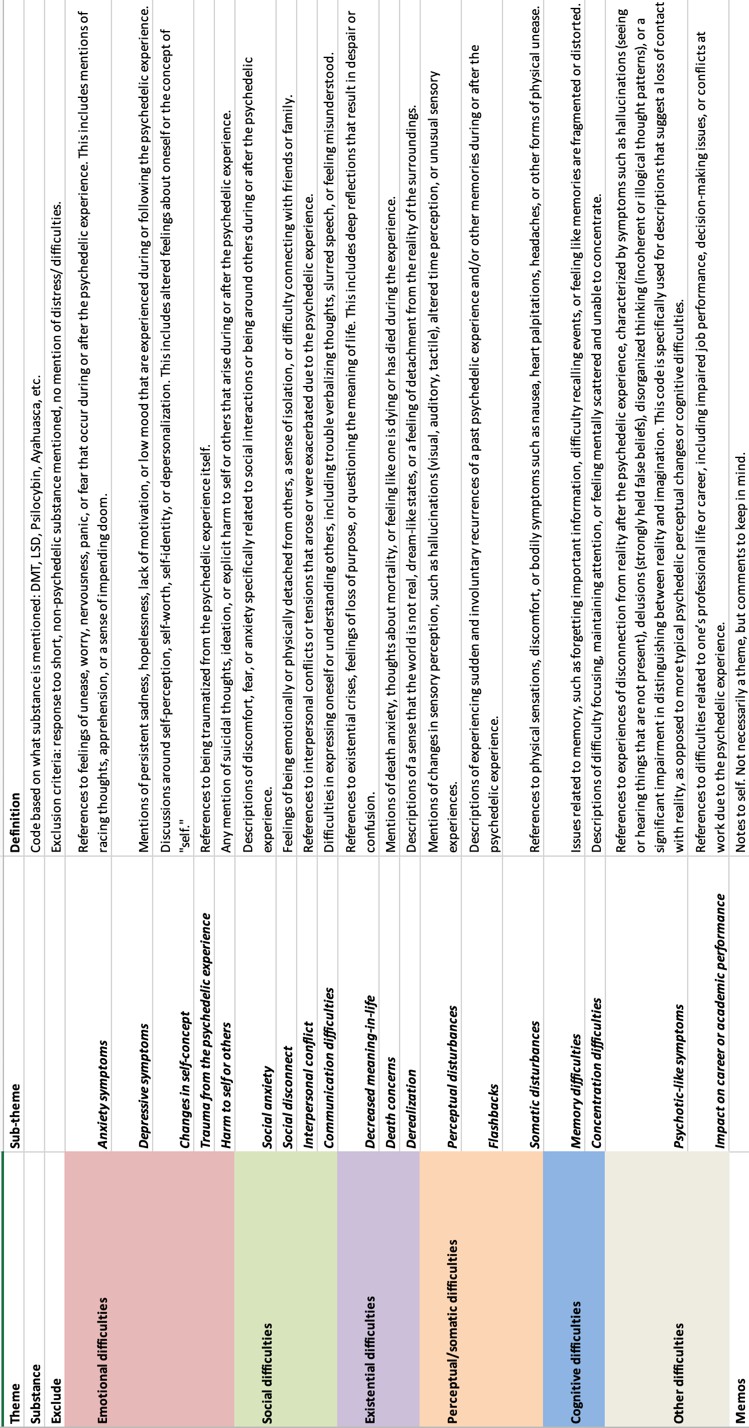


**Figure S2.** **Sample Selection and Exclusion Flowchart.** This figure illustrates the sample selection process, detailing the sub-sampling of psychedelic users from the larger study, as well as the number of participants who reported difficulties and were included in qualitative analyses.


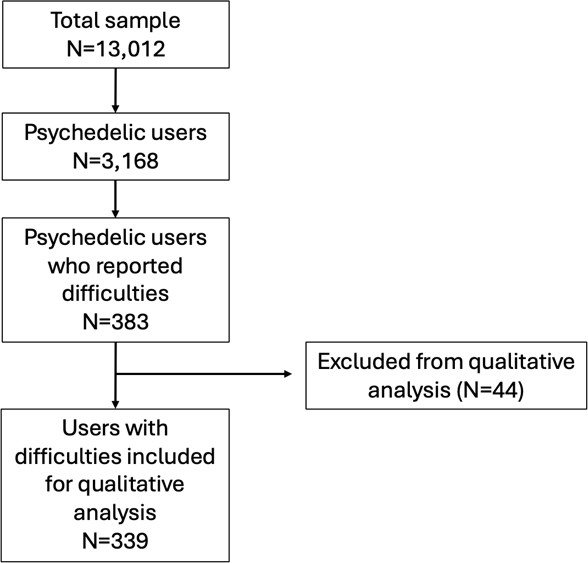


**Figure S3. Qualitative Theme Heatmap**. Depicts overlap of reporting themes among qualitative responses, represented in percentages.


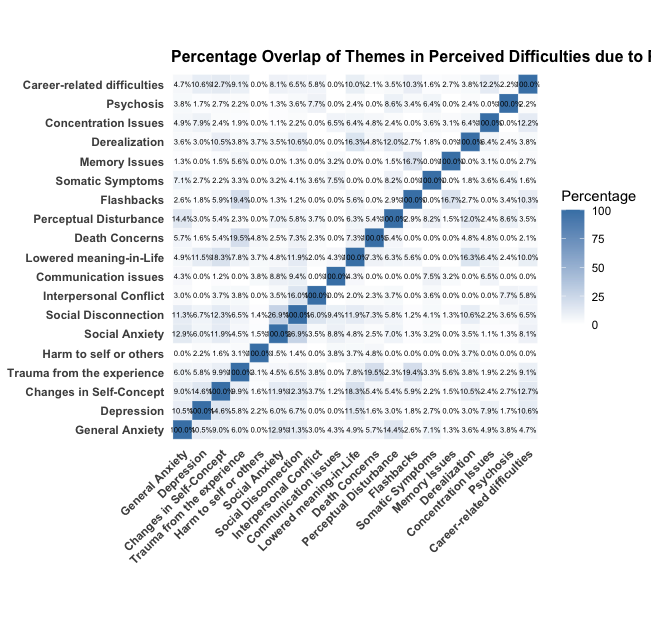


**Figure S4.** **Themes by Psychedelic Group.** Frequencies of sub-themes among qualitative reports (n=339) by psychedelic use group.


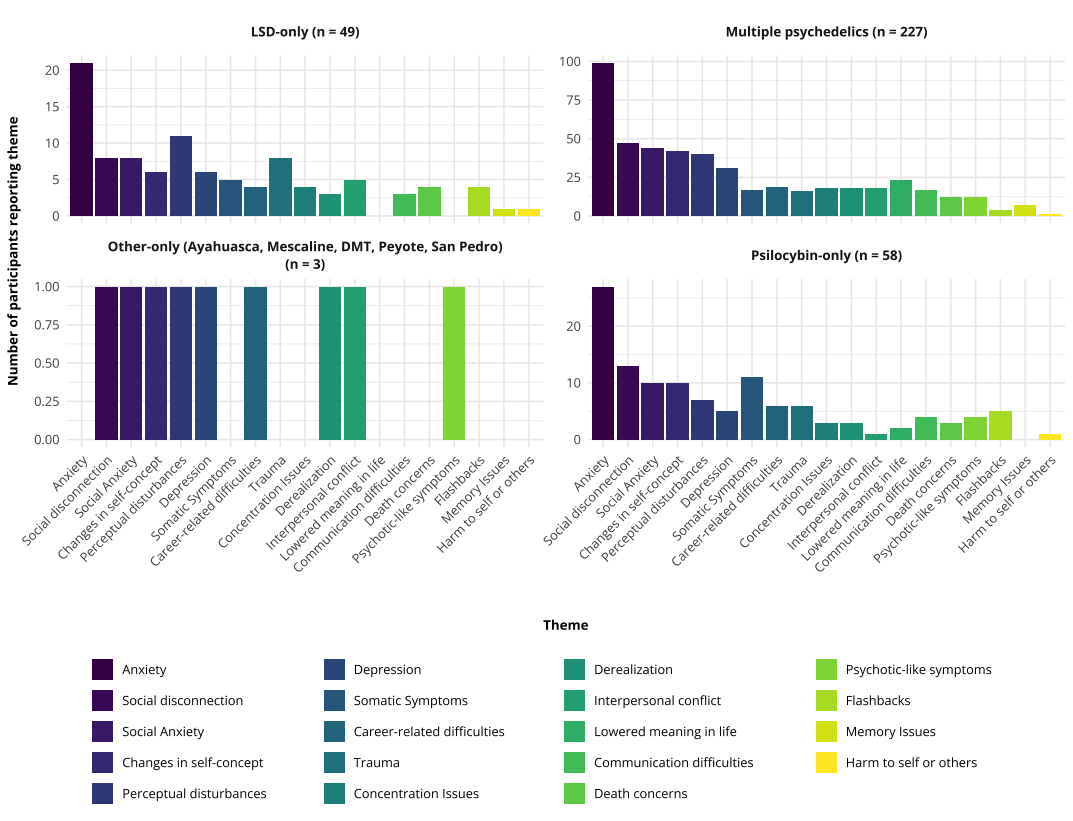


### Table S1. Sample Characteristics of ACEs. Illustrates additional characteristics of ACEs in the total sample (n=3,168), including total score, categorical ACE breakdown, and specific ACE item frequencies.

| **Characteristic** | **N = 3,168**^1^ |
| --- | --- |
| **ACE Total Score (0–8)** | 3.57 (2.22) |
| **ACE Categorical (0,1,2,3,≥4)** |  |
| 0 | 286 (9.0%) |
| 1 | 386 (12.2%) |
| 2 | 443 (14.0%) |
| 3 | 463 (14.6%) |
| 4+ | 1,590 (50.2%) |
| **ACE1. Familial mental illness** | 1,928 (60.9%) |
| **ACE2. Familial alcoholism** | 1,444 (45.6%) |
| **ACE3. Familial drug abuse** | 1,112 (35.1%) |
| **ACE4. Familial incarceration** | 666 (21.0%) |
| **ACE5. Parental divorce** | 1,498 (47.3%) |
| **ACE6. Household violence** | 951 (30.0%) |
| **ACE7. Physical abuse** | 1,513 (47.8%) |
| **ACE8. Verbal abuse** | 2,160 (68.2%) |
| **ACE9. Sexual abuse (touched sexually)** | 779 (24.6%) |
| **ACE10. Sexual abuse (forced to touch sexually)** | 612 (19.3%) |
| **ACE11. Sexual abuse (forced to have sex)** | 357 (11.3%) |
| ^1^Mean (SD); n (%) | |

**Table S2.** **Frequency of Psychedelic-Related Difficulties by Psychedelic Group.** This table illustrates differences in the frequency of reporting difficulties by psychedelic use group (e.g., LSD-only, multiple psychedelics, other-only (ayahuasca, mescaline, DMT, peyote, san pedro), or psilocybin-only).

| **Characteristic** | **Difficulties**  N = 383^1^ | **No Difficulties**  N = 2,785^1^ | **p-value**^2^ |
| --- | --- | --- | --- |
| Psychedelic group |  |  | <0.001 |
| LSD-only | 55 (10.2%) | 482 (89.8%) |  |
| Multiple psychedelics | 260 (15.9%) | 1,379 (84.1%) |  |
| Other-only (Ayahuasca, Mescaline, DMT, Peyote, San Pedro) | 5 (7.8%) | 59 (92.2%) |  |
| Psilocybin-only | 63 (6.8%) | 865 (93.2%) |  |
| ^1^n (row %) | | | |
| ^2^Pearson's Chi-squared test | | | |

**Table S3. Duration of Pychedelic-Related Difficulties by Psychedelic Group.** Illustrates differences in frequency of psychedelic-related difficulties by psychedelic use group (e.g., LSD-only, multiple psychedelics, other-only (ayahuasca, mescaline, DMT, peyote, san pedro), or psilocybin-only).

| **Characteristic** | **No difficulties  N = 2,785^1^** | **≤1 day  N = 180^1^** | **A few days – 1 week  N = 87^1^** | **>1 week – 1 month  N = 45^1^** | **>1 month – 1 year  N = 31^1^** | **>1 year  N = 40^1^** | **p-value^2^** |
| --- | --- | --- | --- | --- | --- | --- | --- |
| **All psychedelic users** | 2,785 (87.9%) | 180 (5.7%) | 87 (2.7%) | 45 (1.4%) | 31 (1.0%) | 40 (1.3%) |  |
| **Psychedelic Group** |  |  |  |  |  |  | <0.001 |
| Multiple psychedelics | 1,379 (84.1%) | 119 (7.3%) | 60 (3.7%) | 30 (1.8%) | 25 (1.5%) | 26 (1.6%) |  |
| LSD-only | 482 (89.8%) | 20 (3.7%) | 15 (2.8%) | 9 (1.7%) | 2 (0.4%) | 9 (1.7%) |  |
| Psilocybin-only | 865 (93.2%) | 40 (4.3%) | 11 (1.2%) | 4 (0.4%) | 3 (0.3%) | 5 (0.5%) |  |
| Other-only (Ayahuasca, Mescaline, DMT, Peyote, San Pedro) | 59 (92.2%) | 1 (1.6%) | 1 (1.6%) | 2 (3.1%) | 1 (1.6%) | 0 (0%) |  |
| ^1^n (row %) | | | | | | | |
| ^2^Pearson's Chi-squared test | | | | | | | |

**Table S4. Summary table of theme frequencies and relevant citations.**

| **Theme** | **Sub-theme** | | **N = 339** | **Description** | **Example** |
| --- | --- | --- | --- | --- | --- |
| **Emotional difficulties** |  | | 282 (83.2%) |  |  |
|  | *General anxiety* | | 147 (43.4%) | References to feelings of unease, worry, nervousness, panic, or fear. | *“Prior to using psychedelics I never had panic attacks…since then it comes and goes.”*  *(Reported duration: a few days to 1 week)* |
|  | *Depression* | | 43 (12.7%) | Mentions of persistent sadness, hopelessness, lack of motivation, or low mood. | *“It made me more depressed than usual.”*  *(Reported duration: More than 1 year)* |
|  | *Changes in self-concept* | | 59 (17.4%) | Discussions around self-perception, self-worth, self-identity, or depersonalization. This includes altered feelings about oneself or the concept of "self." | *“I obsessed over every failing I’ve ever had”*  *(Reported duration: a few days to 1 week)* |
|  | *Trauma from the experience* | | 30 (8.8%) | References to being traumatized from the psychedelic experience itself. | *“Had PTSD. I believed that I died during that trip and that I had never came back to real life.”*  *(Reported duration: more than 1 year)* |
|  | *Harm to self or others* | | 3 (0.9%) | Any mention of suicidal thoughts, ideation, or explicit harm to self or others. | *“It was so distressing that I considered suicide.”*  *(Reported duration: more than 1 year)* |
| **Social difficulties** |  | | 181 (53.4%) |  |  |
|  | *Social anxiety* | | 63 (18.6%) | Descriptions of discomfort, fear, or anxiety specifically related to social interactions | *“That same trip really completely shattered me. People looked different, I felt different. I've had problems with social anxiety ever since then really.”*  *(Reported duration: more than 1 year)* |
|  | *Social disconnection* | | 69 (20.4%) | Feelings of being emotionally or physically detached from others, a sense of isolation, or difficulty connecting with friends or family. | *“I felt that I was just vibrating and connecting with everything on a different level and others just did not understand because we were not on the same wave length.”*  *(Reported duration: more than 1 month to 1 year)* |
|  | *Interpersonal conflict* | | 25 (7.4%) | References to interpersonal conflicts or tensions that arose or were exacerbated due to the psychedelic experience. | *“Ended up having interpersonal difficulties after realizing social games.”*  *(Reported duration: more than 1 week to 1 month)* |
|  | *Communication difficulties* | | 24 (7.1%) | Difficulties in expressing oneself or understanding others, including trouble verbalizing thoughts, slurred speech, or feeling misunderstood. | *“I was unable to speak correctly.”*  *(Reported duration: a few days to 1 week)* |
| **Existential difficulties** |  | | 69 (20.4%) |  |  |
|  | *Lowered meaning in life* | | 25 (7.4%) | References to existential crises, feelings of loss of purpose, or questioning the meaning of life. | *“It made me realize that if it's up to me how I want to frame reality then I'm not sure I even have the willpower.”*  *(Reported duration: a few days to 1 week)* |
|  | *Death concerns* | | 19 (5.6%) | Mentions of death anxiety, thoughts about mortality, or feeling like one is dying or has died during the experience. | *“It gave me an existential crisis which lead to having a fear of death for AT LEAST 3 years that I can confidently say where my every waking hour I was consumed with the thought of dying.”*  *(Reported duration: more than 1 year)* |
|  | *Derealization* | | 25 (7.4%) | Descriptions of a sense that the world is not real, dream-like states, or a feeling of detachment from the reality of the surroundings. | *“I had difficulty with knowing if what was happening was real.”*  *(Reported duration: more than 1 month to 1 year)* |
| **Perceptual & Somatic difficulties** |  | | 105 (31.0%) |  |  |
|  | *Perceptual disturbance* | | 59 (17.4%) | Mentions of changes in sensory perception, such as hallucinations (visual, auditory, tactile), altered time perception, or unusual sensory experiences. | *“I had to go to rehab and I still see visuals on the daily”*  *(Reported duration: more than 1 year)* |
|  | *Flashbacks* | | 13 (3.8%) | Descriptions of experiencing sudden and involuntary recurrences of a past psychedelic experience or other memories. | *“I was nervous to drive for many years after since I had a flashback while driving. It’s been nine years since the experience and only in the last couple of years do I feel that I have recovered from the experience.”*  *(Reported duration: more than 1 year)* |
|  | *Somatic symptoms* | | 33 (9.7%) | References to physical sensations, discomfort, or bodily symptoms such as nausea, heart palpitations, headaches, or other forms of physical unease. | *“Physical numbness, vomiting, overheating, sleeplessness, confusion.”*  *(Reported duration: a few days to 1 week)* |
| **Cognitive difficulties** |  | | 33 (9.7%) |  |  |
|  | *Memory issues* | | 8 (2.4%) | Issues related to memory, such as forgetting important information, difficulty recalling events, or feeling like memories are fragmented or distorted. | *“I was having large gaps in my memory. Forgetting who I am and where I have been..” (Reported duration: more than 1 month to 1 year)* |
|  | *Concentration issues* | | 25 (7.4%) | Descriptions of difficulty focusing, maintaining attention, or feeling mentally scattered and unable to concentrate. | “*For a few days after use I was having a hard time keeping up with conversations. It felt as if my brain had slowed down to half speed and everyone else's was going too fast to understand.”*  *(Reported duration: a few days to 1 week)* |
| **Other difficulties** |  | | 47 (13.9%) |  |  |
|  | *Psychotic-like symptoms* | | 17 (5.0%) | References to experiences of disconnection from reality after the psychedelic experience characterized by symptoms such as hallucinations (seeing or hearing things that are not present), delusions (strongly held false beliefs), disorganized thinking (incoherent or illogical thought patterns), or a significant impairment in distinguishing between reality and imagination. | *“Felt like I was losing my mind because I thought I was telepathic.”*  *(Reported duration: more than 1 week to 1 month)* |
|  | *Career-related difficulties* | | 30 (8.8%) | References to difficulties related to one’s professional life or career, including impaired job performance, decision-making issues, or conflicts at work due to the psychedelic experience. | *“I had a lot of work anxiety and did not want to get on calls or go back into my office. extreme dread.”*  *(Reported duration: a few days to 1 week)* |
|  |  | ^1^n (%) |  |  |  |

**Table S5. Theme Frequencies Across Acute and Post-Acute Difficulties.** Frequencies of themes and sub-themes stratified by reporting of difficulties as ≤1 day or > 1 day.

| **Main Theme** | **Sub-theme** | **Difficulties**  **≤1 day** | **Difficulties**  **> 1 day p-value**^2^ | |
| --- | --- | --- | --- | --- |
|  |  | N = 165^1^ | N = 174^1^ |  |
| Emotional Difficulties |  |  |  |  |
|  | General Anxiety | 88 (53.3%) | 59 (33.9%) | <0.001 |
|  | Depression | 13 (7.9%) | 30 (17.2%) | 0.014 |
|  | Changes in self-concept | 14 (8.5%) | 45 (25.9%) | <0.001 |
|  | Trauma from the experience | 5 (3.0%) | 25 (14.4%) | <0.001 |
|  | Harm to self or others | 1 (0.6%) | 2 (1.1%) | >0.999 |
| Social difficulties |  |  |  |  |
|  | Social anxiety | 35 (21.2%) | 28 (16.1%) | 0.264 |
|  | Social disconnection | 29 (17.6%) | 40 (23.0%) | 0.227 |
|  | Interpersonal conflict | 6 (3.6%) | 19 (10.9%) | 0.012 |
|  | Communication difficulties | 19 (11.5%) | 5 (2.9%) | 0.002 |
| Existential difficulties |  |  |  |  |
|  | Lowered meaning in life | 6 (3.6%) | 19 (10.9%) | 0.012 |
|  | Death concerns | 7 (4.2%) | 12 (6.9%) | 0.349 |

|  | Derealization | 9 (5.5%) | 16 (9.2%) | 0.216 |
| --- | --- | --- | --- | --- |
| Perceptual & Somatic difficulties |  |  |  |  |
|  | Perceptual disturbance | 31 (18.8%) | 28 (16.1%) | 0.567 |
|  | Flashbacks | 4 (2.4%) | 9 (5.2%) | 0.260 |
|  | Somatic Symptoms | 24 (14.5%) | 9 (5.2%) | 0.005 |
| Cognitive difficulties |  |  |  |  |
|  | Memory Issues | 5 (3.0%) | 3 (1.7%) | 0.492 |
|  | Concentration Issues | 14 (8.5%) | 11 (6.3%) | 0.535 |
| Other difficulties |  |  |  |  |
|  | Psychotic-like symptoms | 7 (4.2%) | 10 (5.7%) | 0.622 |
|  | Career-related difficulties | 1 (0.6%) | 29 (16.7%) | <0.001 |
| ^1^n (%) |  |  |  |  |
| ^2^Fisher's exact test |  |  |  |  |

### Table S6. Theme Frequencies by Duration of Psychedelic-Related Difficulties. Details the frequencies of themes and sub-themes stratified by specific reported duration.

| **Main theme** | **Sub-theme** | **1 day or less**  N = 165^1^ | **For a few days to 1 week**  N = 77^1^ | **More than 1 week to 1 month**  N = 39^1^ | **More than 1 month to 1 year**  N = 24^1^ | **More than 1 year**  N = 34^1^ | **p-value**^2^ |
| --- | --- | --- | --- | --- | --- | --- | --- |
| Emotional Difficulties |  |  |  |  |  |  |  |
|  | General Anxiety | 88 (53.3%) | 26 (33.8%) | 11 (28.2%) | 7 (29.2%) | 15 (44.1%) | 0.004 |
|  | Depression | 13 (7.9%) | 13 (16.9%) | 7 (17.9%) | 5 (20.8%) | 5 (14.7%) | 0.082 |
|  | Changes in self-concept | 14 (8.5%) | 22 (28.6%) | 5 (12.8%) | 10 (41.7%) | 8 (23.5%) | <0.001 |
|  | Trauma from the experience | 5 (3.0%) | 9 (11.7%) | 4 (10.3%) | 2 (8.3%) | 10 (29.4%) | <0.001 |
|  | Harm to self or others | 1 (0.6%) | 1 (1.3%) | 0 (0.0%) | 0 (0.0%) | 1 (2.9%) | 0.418 |
| Social difficulties |  |  |  |  |  |  |  |
|  | Social anxiety | 35 (21.2%) | 18 (23.4%) | 4 (10.3%) | 3 (12.5%) | 3 (8.8%) | 0.187 |
|  | Social disconnection | 29 (17.6%) | 27 (35.1%) | 9 (23.1%) | 1 (4.2%) | 3 (8.8%) | 0.001 |
|  | Interpersonal conflict | 6 (3.6%) | 13 (16.9%) | 4 (10.3%) | 0 (0.0%) | 2 (5.9%) | 0.004 |
|  | Communication difficulties | 19 (11.5%) | 4 (5.2%) | 1 (2.6%) | 0 (0.0%) | 0 (0.0%) | 0.037 |
| Existential Difficulties |  |  |  |  |  |  |  |
|  | Lowered meaning in life | 6 (3.6%) | 11 (14.3%) | 1 (2.6%) | 3 (12.5%) | 4 (11.8%) | 0.011 |
|  | Death concerns | 7 (4.2%) | 6 (7.8%) | 1 (2.6%) | 0 (0.0%) | 5 (14.7%) | 0.095 |
|  | Derealization | 9 (5.5%) | 4 (5.2%) | 4 (10.3%) | 3 (12.5%) | 5 (14.7%) | 0.182 |
|  |  |  |  |  |  |  |  |
|  | Perceptual disturbance | 31 (18.8%) | 12 (15.6%) | 5 (12.8%) | 5 (20.8%) | 6 (17.6%) | 0.888 |
|  | Flashbacks | 4 (2.4%) | 2 (2.6%) | 2 (5.1%) | 1 (4.2%) | 4 (11.8%) | 0.112 |
| Perceptual & Somatic Difficutlies |  |  |  |  |  |  |  |
|  | Somatic Symptoms | 24 (14.5%) | 6 (7.8%) | 1 (2.6%) | 2 (8.3%) | 0 (0.0%) | 0.024 |
|  | Memory Issues | 5 (3.0%) | 0 (0.0%) | 2 (5.1%) | 1 (4.2%) | 0 (0.0%) | 0.210 |
|  | Concentration Issues | 14 (8.5%) | 6 (7.8%) | 4 (10.3%) | 0 (0.0%) | 1 (2.9%) | 0.518 |
| Other Difficulties |  |  |  |  |  |  |  |
|  | Psychotic-like symptoms | 7 (4.2%) | 5 (6.5%) | 1 (2.6%) | 2 (8.3%) | 2 (5.9%) | 0.717 |
|  | Career-related difficulties | 1 (0.6%) | 10 (13.0%) | 5 (12.8%) | 6 (25.0%) | 8 (23.5%) | <0.001 |
| ^1^n (%) |  | | | | | | |
| ^2^Fisher's exact test |  | | | | | | |

**Table S7. Full Model Output for Main Text Table 4.** Summary of all covariates from the final models presented in Table 4 of the main text.

|  | Any Difficulties | | | > 1 Day | | | > 1 Week | | | > 1 Month | | | > 1 Year | | |
| --- | --- | --- | --- | --- | --- | --- | --- | --- | --- | --- | --- | --- | --- | --- | --- |
| **Characteristic** | **aOR**^1^ | **95% CI**^1^ | **p-value** | **aOR**^1^ | **95% CI**^1^ | **p-value** | **aOR**^1^ | **95% CI**^1^ | **p-value** | **aOR**^1^ | **95% CI**^1^ | **p-value** | **aOR**^1^ | **95% CI**^1^ | **p-value** |
| ACEs |  |  |  |  |  |  |  |  |  |  |  |  |  |  |  |
| 0 | — | — |  | — | — |  | — | — |  | — | — |  | — | — |  |
| 1 | 1.33 | 0.71, 2.58 | 0.378 | 1.44 | 0.65, 3.42 | 0.385 | 2.72 | 0.97, 9.66 | 0.080 | 2.93 | 0.72, 19.6 | 0.177 | 2.22 | 0.28, 45.2 | 0.491 |
| 2 | 2.24 | 1.27, 4.16 | 0.007 | 1.97 | 0.94, 4.52 | 0.086 | 2.43 | 0.87, 8.59 | 0.118 | 2.41 | 0.59, 16.1 | 0.270 | 2.96 | 0.47, 57.2 | 0.324 |
| 3 | 2.27 | 1.29, 4.20 | 0.006 | 2.14 | 1.03, 4.88 | 0.053 | 2.15 | 0.75, 7.68 | 0.185 | 2.46 | 0.61, 16.5 | 0.259 | 3.67 | 0.62, 69.9 | 0.232 |
| ≥4 | 2.84 | 1.72, 5.03 | <0.001 | 2.37 | 1.25, 5.12 | 0.015 | 2.89 | 1.17, 9.63 | 0.042 | 3.89 | 1.18, 24.0 | 0.063 | 4.36 | 0.90, 78.5 | 0.152 |
| Gender |  |  |  |  |  |  |  |  |  |  |  |  |  |  |  |
| Male | — | — |  | — | — |  | — | — |  | — | — |  | — | — |  |
| Female | 0.74 | 0.59, 0.94 | 0.012 | 0.71 | 0.52, 0.97 | 0.032 | 0.73 | 0.49, 1.09 | 0.126 | 0.68 | 0.40, 1.14 | 0.147 | 0.80 | 0.41, 1.57 | 0.510 |
| Other | 0.64 | 0.40, 0.99 | 0.052 | 0.76 | 0.42, 1.30 | 0.334 | 0.87 | 0.41, 1.69 | 0.702 | 1.04 | 0.41, 2.30 | 0.922 | 0.91 | 0.21, 2.78 | 0.883 |
| Age |  |  |  |  |  |  |  |  |  |  |  |  |  |  |  |
| 18-24 | — | — |  | — | — |  | — | — |  | — | — |  | — | — |  |
| 25-34 | 0.67 | 0.51, 0.88 | 0.004 | 0.71 | 0.50, 1.03 | 0.063 | 0.60 | 0.38, 0.96 | 0.031 | 0.66 | 0.36, 1.27 | 0.202 | 0.80 | 0.33, 2.13 | 0.632 |
| 35-44 | 0.41 | 0.30, 0.58 | <0.001 | 0.37 | 0.23, 0.58 | <0.001 | 0.35 | 0.19, 0.63 | <0.001 | 0.62 | 0.31, 1.27 | 0.186 | 1.19 | 0.48, 3.22 | 0.721 |
| 45-50 | 0.26 | 0.15, 0.42 | <0.001 | 0.36 | 0.18, 0.65 | 0.001 | 0.46 | 0.21, 0.92 | 0.035 | 0.58 | 0.21, 1.43 | 0.254 | 0.85 | 0.22, 2.95 | 0.809 |
| Frequency of Psychedelic Use |  |  |  |  |  |  |  |  |  |  |  |  |  |  |  |
| 1 | — | — |  | — | — |  | — | — |  | — | — |  | — | — |  |
| 2-5 | 1.96 | 1.26, 3.16 | 0.004 | 1.09 | 0.64, 1.95 | 0.747 | 1.93 | 0.90, 4.79 | 0.116 | 2.14 | 0.81, 7.34 | 0.164 | 1.69 | 0.55, 7.36 | 0.414 |
| 6-10 | 2.53 | 1.59, 4.18 | <0.001 | 1.83 | 1.05, 3.30 | 0.038 | 1.82 | 0.77, 4.76 | 0.192 | 1.39 | 0.43, 5.27 | 0.592 | 0.68 | 0.12, 3.70 | 0.637 |
| 11-20 | 3.30 | 2.04, 5.50 | <0.001 | 2.12 | 1.19, 3.90 | 0.013 | 3.30 | 1.44, 8.50 | 0.007 | 3.32 | 1.16, 11.9 | 0.038 | 2.58 | 0.73, 11.9 | 0.166 |
| 21-50 | 4.24 | 2.55, 7.24 | <0.001 | 3.08 | 1.69, 5.80 | <0.001 | 5.46 | 2.37, 14.2 | <0.001 | 4.40 | 1.49, 16.1 | 0.012 | 3.10 | 0.83, 14.7 | 0.109 |
| 51-100 | 3.62 | 1.86, 6.98 | <0.001 | 1.79 | 0.71, 4.19 | 0.192 | 3.53 | 1.09, 11.1 | 0.029 | 5.37 | 1.47, 21.8 | 0.012 | 4.33 | 0.91, 22.8 | 0.063 |
| 101-300 | 5.85 | 2.67, 12.4 | <0.001 | 3.57 | 1.31, 8.87 | 0.008 | 6.21 | 1.74, 20.7 | 0.003 | 5.35 | 1.01, 25.6 | 0.034 | 0.00 | NE^2^ | 0.987 |
| 300+ | 6.43 | 2.56, 15.2 | <0.001 | 4.97 | 1.66, 13.2 | 0.002 | 6.04 | 1.23, 23.5 | 0.013 | 5.77 | 0.77, 31.5 | 0.051 | 3.40 | 0.16, 28.4 | 0.301 |
| ^1^aOR = Adjusted Odds Ratio, CI = Confidence Interval  ^2^Not estimable due to perfect separation at this predictor level (i.e., 0 counts in one cell). A sensitivity analysis using Firth’s penalized regression was performed. The direction and magnitude of results were broadly similar. | | | | | | | | | | | | | | | |

**Table S8. Sensitivity Analysis with ACE Total Score.** Full model outputs from logistic regression using ACE total score.

|  | Any Difficulties | | | > 1 Day | | | > 1 Week | | | > 1 Month | | | > 1 Year | | |
| --- | --- | --- | --- | --- | --- | --- | --- | --- | --- | --- | --- | --- | --- | --- | --- |
| **Characteristic** | **aOR**^1^ | **95% CI**^1^ | **p-value** | **aOR**^1^ | **95% CI**^1^ | **p-value** | **aOR**^1^ | **95% CI**^1^ | **p-value** | **aOR**^1^ | **95% CI**^1^ | **p-value** | **aOR**^1^ | **95% CI**^1^ | **p-value** |
| ACE Total Score | 1.13 | 1.08, 1.19 | <0.001 | 1.12 | 1.05, 1.20 | <0.001 | 1.09 | 1.00, 1.19 | 0.052 | 1.17 | 1.05, 1.31 | 0.004 | 1.17 | 1.01, 1.35 | 0.033 |
| Gender |  |  |  |  |  |  |  |  |  |  |  |  |  |  |  |
| Male | — | — |  | — | — |  | — | — |  | — | — |  | — | — |  |
| Female | 0.74 | 0.59, 0.93 | 0.011 | 0.71 | 0.52, 0.96 | 0.028 | 0.73 | 0.48, 1.09 | 0.120 | 0.66 | 0.39, 1.11 | 0.121 | 0.79 | 0.40, 1.55 | 0.491 |
| Other | 0.62 | 0.39, 0.97 | 0.043 | 0.73 | 0.40, 1.25 | 0.276 | 0.85 | 0.39, 1.66 | 0.649 | 0.97 | 0.38, 2.16 | 0.952 | 0.88 | 0.20, 2.71 | 0.846 |
| Age |  |  |  |  |  |  |  |  |  |  |  |  |  |  |  |
| 18-24 | — | — |  | — | — |  | — | — |  | — | — |  | — | — |  |
| 25-34 | 0.66 | 0.50, 0.87 | 0.003 | 0.70 | 0.49, 1.00 | 0.048 | 0.59 | 0.37, 0.95 | 0.027 | 0.65 | 0.35, 1.25 | 0.186 | 0.78 | 0.32, 2.07 | 0.593 |
| 35-44 | 0.41 | 0.29, 0.57 | <0.001 | 0.36 | 0.23, 0.57 | <0.001 | 0.34 | 0.19, 0.61 | <0.001 | 0.60 | 0.30, 1.24 | 0.164 | 1.15 | 0.46, 3.14 | 0.765 |
| 45-50 | 0.25 | 0.15, 0.41 | <0.001 | 0.35 | 0.18, 0.64 | 0.001 | 0.45 | 0.21, 0.91 | 0.033 | 0.57 | 0.21, 1.40 | 0.240 | 0.84 | 0.21, 2.90 | 0.786 |
| Frequency of Psychedelic Use |  |  |  |  |  |  |  |  |  |  |  |  |  |  |  |
| 1 | — | — |  | — | — |  | — | — |  | — | — |  | — | — |  |
| 2-5 | 1.92 | 1.24, 3.10 | 0.005 | 1.07 | 0.63, 1.91 | 0.800 | 1.89 | 0.88, 4.68 | 0.129 | 2.08 | 0.79, 7.14 | 0.180 | 1.66 | 0.54, 7.23 | 0.430 |
| 6-10 | 2.48 | 1.56, 4.10 | <0.001 | 1.79 | 1.03, 3.23 | 0.046 | 1.79 | 0.76, 4.70 | 0.201 | 1.36 | 0.42, 5.14 | 0.621 | 0.66 | 0.12, 3.62 | 0.617 |
| 11-20 | 3.21 | 1.99, 5.36 | <0.001 | 2.06 | 1.15, 3.81 | 0.017 | 3.26 | 1.42, 8.40 | 0.008 | 3.20 | 1.11, 11.5 | 0.045 | 2.49 | 0.71, 11.5 | 0.183 |
| 21-50 | 4.17 | 2.50, 7.12 | <0.001 | 3.01 | 1.64, 5.66 | <0.001 | 5.39 | 2.34, 14.0 | <0.001 | 4.23 | 1.43, 15.5 | 0.015 | 2.99 | 0.80, 14.2 | 0.121 |
| 51-100 | 3.53 | 1.81, 6.83 | <0.001 | 1.73 | 0.68, 4.06 | 0.219 | 3.47 | 1.07, 10.9 | 0.031 | 5.13 | 1.40, 20.8 | 0.014 | 4.20 | 0.89, 22.2 | 0.069 |
| 101-300 | 5.67 | 2.60, 12.0 | <0.001 | 3.47 | 1.27, 8.61 | 0.010 | 6.19 | 1.74, 20.6 | 0.003 | 5.27 | 0.99, 25.2 | 0.036 | 0.00 | NE^2^ | 0.987 |
| 300+ | 6.07 | 2.41, 14.4 | <0.001 | 4.70 | 1.57, 12.5 | 0.003 | 5.66 | 1.15, 22.1 | 0.017 | 5.09 | 0.67, 28.0 | 0.071 | 3.09 | 0.15, 26.1 | 0.342 |
| ^1^OR = Odds Ratio, CI = Confidence Interval  ^2^Not estimable due to perfect separation at this predictor level (i.e., 0 counts in one cell). A sensitivity analysis using Firth’s penalized regression was performed. The direction and magnitude of results were broadly similar. | | | | | | | | | | | | | | | |

**Table S9. Frequencies of difficulties by ACE group.** Cross-tabulated distribution of difficulties by ACE categorical group.

| ACE Categorical | No difficulties | | Any difficulties | >1 Day | > 1 week | > 1 month | > 1 year |
| --- | --- | --- | --- | --- | --- | --- | --- |
| 0 | 270 (9.7%) | | 16 (4.1%) | 9 (4.4%) | 4 (3.4%) | 2 (2.8%) | 1 (2.5%) |
| 1 | 356 (12.8%) | | 29 (7.6%) | 18 (8.9%) | 15 (12.9%) | 8 (11.3%) | 3 (7.5%) |
| 2 | 389 (14.0%) | | 53 (13.8%) | 28 (13.8%) | 16 (13.8%) | 8 (11.3%) | 5 (12.5%) |
| 3 | 408 (14.7%) | | 54 (14.1%) | 30 (14.8%) | 14 (12.1%) | 8 (11.3%) | 6 (15.0%) |
| ≥4 | | 1,359 (48.8%) | 231 (60.3%) | 118 (58.1%) | 67 (57.8%) | 45 (63.4%) | 25 (62.5%) |

**Table S10. Logistic Regression on ACE domains.** Full model outputs of models assessing associations between the three main ACE domains (household dysfunction, physical/emotional abuse, and sexual abuse) and psychedelic-related difficulties.

|  | *Any difficulties* | | | *> 1 day* | | | *> 1 week* | | | *> 1 month* | | | *> 1 year* | | |
| --- | --- | --- | --- | --- | --- | --- | --- | --- | --- | --- | --- | --- | --- | --- | --- |
| **Characteristic** | **aOR**^1^ | **95% CI**^1^ | **p-value** | **aOR**^1^ | **95% CI**^1^ | **p-value** | **aOR**^1^ | **95% CI**^1^ | **p-value** | **aOR**^1^ | **95% CI**^1^ | **p-value** | **aOR**^1^ | **95% CI**^1^ | **p-value** |
| Household dysfunction | 1.39 | 1.00, 1.96 | 0.053 | 0.95 | 0.63, 1.45 | 0.791 | 0.68 | 0.41, 1.15 | 0.135 | 0.98 | 0.50, 2.05 | 0.943 | 1.31 | 0.53, 3.99 | 0.593 |
| Physical/emotional abuse | 1.64 | 1.23, 2.23 | 0.001 | 1.69 | 1.14, 2.58 | 0.012 | 1.36 | 0.83, 2.33 | 0.235 | 1.23 | 0.66, 2.43 | 0.538 | 1.28 | 0.57, 3.29 | 0.580 |
| Sexual abuse | 1.43 | 1.12, 1.83 | 0.005 | 1.95 | 1.41, 2.68 | <0.001 | 2.54 | 1.68, 3.84 | <0.001 | 2.36 | 1.40, 3.97 | 0.001 | 1.92 | 0.96, 3.79 | 0.060 |
| Gender |  |  |  |  |  |  |  |  |  |  |  |  |  |  |  |
| Male | — | — |  | — | — |  | — | — |  | — | — |  | — | — |  |
| Female | 0.69 | 0.55, 0.88 | 0.003 | 0.63 | 0.46, 0.86 | 0.004 | 0.62 | 0.40, 0.94 | 0.024 | 0.59 | 0.34, 1.00 | 0.050 | 0.72 | 0.36, 1.44 | 0.348 |
| Other | 0.58 | 0.36, 0.91 | 0.021 | 0.63 | 0.34, 1.10 | 0.119 | 0.70 | 0.32, 1.39 | 0.338 | 0.87 | 0.34, 1.96 | 0.757 | 0.80 | 0.18, 2.51 | 0.736 |
| Age |  |  |  |  |  |  |  |  |  |  |  |  |  |  |  |
| 18-24 | — | — |  | — | — |  | — | — |  | — | — |  | — | — |  |
| 25-34 | 0.66 | 0.50, 0.88 | 0.004 | 0.70 | 0.49, 1.00 | 0.049 | 0.58 | 0.37, 0.94 | 0.023 | 0.65 | 0.35, 1.26 | 0.187 | 0.78 | 0.32, 2.08 | 0.602 |
| 35-44 | 0.41 | 0.29, 0.57 | <0.001 | 0.35 | 0.22, 0.55 | <0.001 | 0.32 | 0.18, 0.58 | <0.001 | 0.58 | 0.29, 1.20 | 0.136 | 1.14 | 0.46, 3.10 | 0.788 |
| 45-50 | 0.25 | 0.14, 0.40 | <0.001 | 0.32 | 0.16, 0.59 | <0.001 | 0.39 | 0.18, 0.79 | 0.012 | 0.51 | 0.19, 1.27 | 0.165 | 0.78 | 0.20, 2.72 | 0.709 |
| Frequency of Psychedelic Use |  |  |  |  |  |  |  |  |  |  |  |  |  |  |  |
| 1 | — | — |  | — | — |  | — | — |  | — | — |  | — | — |  |
| 2-5 | 1.92 | 1.24, 3.10 | 0.005 | 1.05 | 0.62, 1.86 | 0.869 | 1.81 | 0.84, 4.48 | 0.159 | 2.05 | 0.78, 7.03 | 0.189 | 1.64 | 0.53, 7.16 | 0.439 |
| 6-10 | 2.54 | 1.59, 4.19 | <0.001 | 1.78 | 1.02, 3.22 | 0.048 | 1.73 | 0.73, 4.54 | 0.229 | 1.36 | 0.42, 5.14 | 0.621 | 0.67 | 0.12, 3.65 | 0.626 |
| 11-20 | 3.25 | 2.01, 5.43 | <0.001 | 2.02 | 1.13, 3.72 | 0.021 | 3.07 | 1.34, 7.92 | 0.012 | 3.16 | 1.10, 11.4 | 0.047 | 2.50 | 0.71, 11.6 | 0.180 |
| 21-50 | 4.07 | 2.44, 6.96 | <0.001 | 2.82 | 1.54, 5.32 | <0.001 | 4.92 | 2.13, 12.8 | <0.001 | 4.08 | 1.38, 14.9 | 0.017 | 2.92 | 0.78, 13.9 | 0.130 |
| 51-100 | 3.60 | 1.85, 6.96 | <0.001 | 1.77 | 0.70, 4.15 | 0.203 | 3.58 | 1.11, 11.3 | 0.028 | 5.47 | 1.50, 22.2 | 0.011 | 4.38 | 0.93, 23.1 | 0.060 |
| 101-300 | 5.66 | 2.58, 12.0 | <0.001 | 3.28 | 1.20, 8.16 | 0.014 | 5.63 | 1.58, 18.8 | 0.005 | 5.08 | 0.96, 24.3 | 0.040 | 0.00 | NE^2^ | 0.987 |
| 300+ | 6.02 | 2.39, 14.3 | <0.001 | 4.35 | 1.45, 11.6 | 0.005 | 4.91 | 1.00, 19.2 | 0.029 | 4.76 | 0.63, 26.2 | 0.083 | 2.97 | 0.14, 25.0 | 0.360 |
| ^1^OR = Odds Ratio, CI = Confidence Interval  ^2^Not estimable due to perfect separation at this predictor level (i.e., 0 counts in one cell). A sensitivity analysis using Firth’s penalized regression was performed. The direction and magnitude of results were broadly similar. | | | | | | | | | | | | | | | |
